# Supplementary material for: Matrix metalloproteinase 1 modulates invasive behavior of tracheal branches during entry into Drosophila flight muscles
Source: eLife. 2019 Oct 2;8:e48857. doi: 10.7554/eLife.48857 (PMC6795481; doi:10.7554/eLife.48857)
Supplement: Supplementary file 3. [file elife-48857-supp3.docx]

**Statistics Reporting Table**

**Figure 1**

**Tracheal terminal cell branches occupy separate territories in IFMs.**

| **Figure Panel** | **Test** | **S.D. or S.E.M.** | **n value** | **Number of times experiment was replicated in laboratory** | **P value** |
| --- | --- | --- | --- | --- | --- |
| 1A,A’ | - | - | n=18 thorax halves | 5 independent experiments | - |
| 1B,B’ | - | - | n=5 thorax halves | 2 independent experiments | - |
| 1C,C’;C’’,C’’’ | - | - | n=14 myotubes of individual thorax halves | 2 independent experiments | - |
| 1D,D’,D’’,D’’’ | - | - | n=5 myotubes of individual thorax halves | The experiment was performed once | - |
| 1E,E’ | - | - | n=31 MARCM clones | 15 independent experiments | - |
| 1F | - | - | n=31 MARCM clones | 15 independent experiments | - |
| 1G | - | - | n=31 MARCM clones | 15 independent experiments | - |
| 1H,H’ | - | - | n=10 myotubes of individual thorax halves | 2 independent experiments | - |

**Figure 2**

**IFM innervation precedes tracheal invasion.**

| **Figure Panel** | **Test** | **S.D. or S.E.M.** | **n value** | **Number of times experiment was replicated in laboratory** | **P value** |
| --- | --- | --- | --- | --- | --- |
| 2A,A’,A’’ | **-** | **-** | n=3 myotubes of individual thorax halves | The experiment was performed once | **-** |
| 2B,B’,B’’ | **-** | **-** | n=3 myotubes of individual thorax halves | The experiment was performed once | **-** |

**Figure 3**

**Tracheal branch invasion requires Mmp1 function in tracheal cells.**

| **Figure Panel** | **Test** | **S.D. or S.E.M.** | **n value** | **Number of times experiment was replicated in laboratory** | **P value** |
| --- | --- | --- | --- | --- | --- |
| 3A,A’ | - | - | n=12 thorax halves | 2 independent experiments | - |
| 3B,B’ | - | - | n=12 thorax halves | 3 independent experiments | - |
| 3C,C’ | - | - | n=11 thorax halves | The experiment was performed once | - |
| 3D,D’ | - | - | n=3 thorax halves | 4 independent experiments | - |
| 3E | - | - | n=2 thorax halves | 2 independent experiments | - |
| 3F | - | - | n=4 thorax halves | 2 independent experiments | - |
| 3G | - | - | n=4-tracheal branch trees of control pupae; n=6 tracheal branch trees for *mmp1* knock-down pupae | 2 independent experiments | - |
| 3H,H’ | - | - | n=43 myotubes of individual thorax halves | 6 independent experiments | - |
| 3I,I’ | - | - | n=22 myotubes of individual thorax halves | 6 independent experiments | - |
| 3J,J’ | - | - | n=28 myotubes of individual thorax halves | The experiment was performed once | - |
| 3K,K’ | - | - | n=45 myotubes of individual thorax halves | 4 independent experiments | - |
| 3L | Kolmogorov-Smirnov-Test | box plot with S.D. | The minimum sample size was n=22 and the maximum n=45 myotubes of individual thorax halves |  | yes |
| 3M | Kolmogorov-Smirnov-Test | box plot with S.D. | The minimum sample size was n=22 and the maximum n=45 myotubes of individual thorax halves |  | yes |
| 3N | Kolmogorov-Smirnov-Test | bar graph with S.D. | n=15-70 flies were subjected to the flight test | 4 independent experiments | yes |

**Figure 4**

**Normal dynamics of tracheal IFM invasion depends on tracheal *mmp1* function.**

| **Figure Panel** | **Test** | **S.D. or S.E.M.** | **n value** | **Number of times experiment was replicated in laboratory** | **P value** |
| --- | --- | --- | --- | --- | --- |
| 4B,B’ | **-** | **-** | n=9 movies of developing pupae | 9 independent experiments | **-** |
| 4C,C’ | **-** | **-** | n=8 movies of developing pupae | 9 independent experiments | **-** |
| 4D,D’ | **-** | **-** | n=5 myotubes of control pupae; n=8 myotubes of *mmp1* knock-down pupae | 3 independent experiments for control pupae; 4 independent experiments for *mmp1* knock-down pupae | **-** |
| 4E | **-** | **-** | n=3 movies of developing pupae | 3 independent experiments | **-** |
| 4F | **-** | **-** | n=3 movies of developing pupae | 3 independent experiments | **-** |

**Figure 5**

**Tracheal stalk and tip regions show distinct basement membrane compositions.**

| **Figure Panel** | **Test** | **S.D. or S.E.M.** | **n value** | **Number of times experiment was replicated in laboratory** | **P value** |
| --- | --- | --- | --- | --- | --- |
| 5A,A’;A’’;A’’’ | **-** | **-** | n=11 myotubes of individual thorax halves | 3 independent experiments | **-** |
| 5B,B’,B’’,B’’’ | **-** | **-** | n=4 myotubes of individual thorax halves | 2 independent experiments | **-** |
| 5C,C’ | **-** | **-** | n=5 myotubes of individual thorax halves | The experiment was performed once | **-** |
| 5D,D’ | **-** | **-** | n=8 myotubes of individual thorax halves | The experiment was performed once | **-** |
| 5E,E’ | **-** | **-** | n=14 myotubes of individual thorax halves | 4 independent experiments | **-** |
| 5F,F’ | **-** | **-** | n=10 myotubes of individual thorax halves | 4 independent experiments were performed using either UAS-*mmp1* RNAi (1) or UAS-*mmp1* RNAi (2) | **-** |

**Figure 1, figure supplement 1**

**Tracheal terminal cells with non-stereotyped cellular morphologies fill the myotube volume.**

| **Figure Panel** | **Test** | **S.D. or S.E.M.** | **n value** | **Number of times experiment was replicated in laboratory** | **P value** |
| --- | --- | --- | --- | --- | --- |
| Supp.1A | **-** | **-** | n=6 | The experiment was performed once |  |
| Supp.1B | **-** | **-** | n=31 wild-type MARCM clones | 15 independent experiments |  |

**Figure 2, figure supplement 1**

**Mitochondria change their morphology during IFM development and partially enwrap tracheoles.**

| **Figure Panel** | **Test** | **S.D. or S.E.M.** | **n value** | **Number of times experiment was replicated in laboratory** | **P value** |
| --- | --- | --- | --- | --- | --- |
| Supp. 2A,A’ | **-** | **-** |  | The experiment was performed once | **-** |
| Supp. 2B,B’ | **-** | **-** |  | The experiment was performed once | **-** |
| Supp. 2C,C’ | **-** | **-** |  | The experiment was performed once | **-** |
| Supp. 2D,D’ | **-** | **-** |  | The experiment was performed once | **-** |
| Supp. 2E,E’ | **-** | **-** |  | The experiment was performed once | **-** |
| Supp. 2F,F’ | **-** | **-** |  | The experiment was performed once | **-** |
| Supp. 2G | **-** | **-** |  | The experiment was performed once | **-** |
| Supp. 2H | **-** | **-** |  | The experiment was performed once | **-** |
| Supp. 2I | **-** | Bar graph shows means with standard deviation | n=3 TEM cross-sections (total of 30 mitochondria, 30 tracheae) were analyzed for 32h APF samples;  n=4 TEM sections (total of 40 mitochondria, 40 tracheae) were analyzed for 48h APF samples;  n=5 TEM sections (total of 47 mitochondria, 4 tracheae) were analyzed for adult samples. | The experiments was performed once | **-** |
| Supp. 2J,J’ | **-** | **-** |  | The experiment was performed once | **-** |
| Supp. 2K,K’ | **-** | **-** |  | The experiment was performed once | **-** |
| Supp. 2L,M | **-** | **-** |  | The experiment was performed once | **-** |

**Figure 3, figure supplement 1**

**Identification of genes required for branch invasion**

| **Figure Panel** | **Test** | **S.D. or S.E.M.** | **n value** | **Number of times experiment was replicated in laboratory** | **P value** |
| --- | --- | --- | --- | --- | --- |
| Supp. 3B,B’,B’’ | **-** | **-** | n=22 myotubes of individual thorax halves | 2 independent experiments | **-** |
| Supp. 3C,C’,C’’ | **-** | **-** | n=20 myotubes of individual thorax halves | 2 independent experiments | **-** |
| Supp. 3D,D’,D’’ | **-** | **-** | n=3 myotubes of individual thorax halves | 2 independent experiments | **-** |
| Supp. 3E,E’ | **-** | **-** | n=3 myotubes of individual thorax halves | 2 independent experiments | **-** |
| Supp. 3F,F’ | **-** | **-** | n=3 myotubes of individual thorax halves | 2 independent experiments | **-** |
| Supp. 3G | Kolmogorov-Smirnov Test | box plot with S.D.**-** | n=2412 mitochondria were analyzed for control myotubes and n=1805 for *bnl* knock-down myotubes | The experiments was performed once | 0,000418 |
| Supp. 3H | Kolmogorov-Smirnov Test | box plot with S.D.**-** | n=2412 mitochondria were analyzed for control myotubes and n=1805 for *bnl* knock-down myotubes | The experiment was performed once | 0,000000885 |

**Figure 4, figure supplement 1**

**MMP1-GFP is distributed along tracheal branches in IFM myotubes.**

| **Figure Panel** | **Test** | **S.D. or S.E.M.** | **n value** | **Number of times experiment was replicated in laboratory** | **P value** |
| --- | --- | --- | --- | --- | --- |
| Supp. 4A,A’,A’’, 4B,B’,B’’ | **-** | **-** | n=10 individual thorax halves | The experiment was performed once | **-** |
| Supp. 4C,C’,C’’, 4D,D’,D’’ | **-** | **-** | n=10 individual thorax halves | The experiment was performed once | **-** |

**Figure 5, figure supplement 1**

**Basement membrane around tracheal branches increases during IFM development.**

| **Figure Panel** | **Test** | **S.D. or S.E.M.** | **n value** | **Number of times experiment was replicated in laboratory** | **P value** |
| --- | --- | --- | --- | --- | --- |
| Supp. 5A,A’ | **-** | **-** | n=7 myotubes of individual thorax halves | The experiment was performed once | **-** |
| Supp. 5B,B’ | **-** | **-** | n=22 myotubes of individual thorax halves | The experiment was performed once | **-** |
| Supp. 5C,C’,C’’ | **-** | **-** | n=11 myotubes of individual thorax halves | 4 independent experiments | **-** |
| Supp. 5D,D’ | **-** | **-** | n=7 myotubes of individual thorax halves | The experiment was performed once | **-** |
| Supp. 5E,E’ | **-** | **-** | n=23 myotubes of individual thorax halves | The experiment was performed once | **-** |
| Supp. 5F,F’ | **-** | **-** | n=8 myotubes of individual thorax halves | The experiment was performed once | **-** |
| Supp. 5G,G’,G’’ | **-** | **-** | n=22 myotubes of individual thorax halves | 6 independent experiments | **-** |
| Supp. 5H,H’,H’’ | **-** | **-** | n=7 myotubes of individual thorax halves | 2 independent experiments | **-** |
